# Multimodal Pyrethroid Resistance in Malaria Vectors, *Anopheles gambiae* s.s., *Anopheles arabiensis*, and *Anopheles funestus* s.s. in Western Kenya

**DOI:** 10.1371/journal.pone.0022574

**Published:** 2011-08-11

**Authors:** Hitoshi Kawada, Gabriel O. Dida, Kazunori Ohashi, Osamu Komagata, Shinji Kasai, Takashi Tomita, George Sonye, Yoshihide Maekawa, Cassian Mwatele, Sammy M. Njenga, Charles Mwandawiro, Noboru Minakawa, Masahiro Takagi

**Affiliations:** 1 Department of Vector Ecology and Environment, Institute of Tropical Medicine, Nagasaki University, Nagasaki, Japan; 2 The Global Center of Excellence Program, Nagasaki University, Nagasaki, Japan; 3 School of Public Health, Maseno University, Kisumu, Kenya; 4 Springs of Hope, Mbita, Kenya; 5 Agricultural Chemicals Research Laboratory, Sumitomo Chemical Co., Ltd., Hyogo, Japan; 6 National Institute of Infectious Diseases, Tokyo, Japan; 7 Eastern and Southern Africa Centre of International Parasite Control, Nairobi, Kenya; 8 Kenya Medical Research Institute, Nairobi, Kenya; Universidade Federal do Rio de Janeiro, Brazil

## Abstract

*Anopheles gambiae* s.s., *Anopheles arabiensis*, and *Anopheles funestus* s.s. are the most important species for malaria transmission. Pyrethroid resistance of these vector mosquitoes is one of the main obstacles against effective vector control. The objective of the present study was to monitor the pyrethroid susceptibility in the 3 major malaria vectors in a highly malaria endemic area in western Kenya and to elucidate the mechanisms of pyrethroid resistance in these species. Gembe East and West, Mbita Division, and 4 main western islands in the Suba district of the Nyanza province in western Kenya were used as the study area. Larval and adult collection and bioassay were conducted, as well as the detection of point mutation in the voltage-gated sodium channel (1014L) by using direct DNA sequencing. A high level of pyrethroid resistance caused by the high frequency of point mutations (L1014S) was detected in *An. gambiae* s.s. In contrast, P450-related pyrethroid resistance seemed to be widespread in both *An. arabiensis* and *An. funestus* s.s. Not a single L1014S mutation was detected in these 2 species. A lack of cross-resistance between DDT and permethrin was also found in *An. arabiensis* and *An. funestus* s.s., while *An. gambiae* s.s. was resistant to both insecticides. It is noteworthy that the above species in the same area are found to be resistant to pyrethroids by their unique resistance mechanisms. Furthermore, it is interesting that 2 different resistance mechanisms have developed in the 2 sibling species in the same area individually. The cross resistance between permethrin and DDT in *An. gambiae* s.s. may be attributed to the high frequency of *kdr* mutation, which might be selected by the frequent exposure to ITNs. Similarly, the metabolic pyrethroid resistance in *An. arabiensis* and *An. funestus* s.s. is thought to develop without strong selection by DDT.

## Introduction

The most exciting event in the history of mosquito control was the invention of dichloro-diphenyl-trichloroethane (DDT). The long persistence, excellent killing efficacy, and strong repellency of DDT are responsible for its considerable success in malaria control after the Second World War. Nevertheless, the first DDT resistance case in *Anopheles* mosquitoes was detected several years later. Furthermore, DDT resistance was responsible for the increase in the incidence of malaria in the 1960s. The number of reported malaria cases throughout the world had increased in 1977 by 2.5 times in comparison with 1973. The development of DDT resistance was one of the main factors caused the above increase [1]. Due to the failure of the malaria eradication program, the World Health Organization (WHO) has altered their policy from eradication to control. Since WHO announced the Roll Back Malaria (RBM) movement in 1998 [2], insecticide-treated nets (ITNs) have become a major tool in the RBM. In Kenya, ITNs have been mainly distributed to pregnant women and children under five years old through programs of the Kenya Ministry of Health and nongovernmental organizations (NGOs) [3], [4]. Consequently, ITN coverage for children under five years old has increased rapidly from 7% in 2004 to 67% in 2006; this increase has been associated with a 44% reduction in malaria deaths [5].

 Pyrethroid is the general term for a group of synthetic chemicals that are structurally modified from natural pyrethrins derived from Chrysanthemum flowers. Most of the pyrethroids are nontoxic to mammals as compared to the insecticide groups and possess high knockdown activity against insects. Nowadays, pyrethroids are emerging as the predominant insecticides for vector control. They are used in various formulations such as long lasting insecticide-treated nets (LLIN) for the long-time prevention of mosquito bites in malaria endemic areas, indoor residual spray, and ultra-low volume (ULV) sprays for emergency control of dengue vectors. In fact, pyrethroids comprise 40% of the insecticides used annually on a global level for indoor residual spraying against malaria vectors and 100% of the WHO-recommended insecticides for the treatment of mosquito nets [6].

The resistance of vector mosquitoes is one of the main obstacles against effective vector control. Pyrethroid resistance is predicted to be a major problem for the vector control program since at present there are no suitable chemical substitutes for pyrethroids. In order to effectively manage pyrethroid resistance, the establishment of a feasible insecticide management system and a regular monitoring system of insecticide susceptibility will be essential. More than 90% of the current annual malaria incidence is found in Africa where the major vectors are *Anopheles gambiae* Giles s.l. that breeds in both temporal stagnant and permanent water pools. Two point mutations at the voltage-gated sodium channel have been found to be associated with knockdown resistance (*kdr*) to DDT and pyrethroids in *An. gambiae*. One mutation involves a leucine (TTA)-phenylalanine (TTT) transversion at residue 1014 of the gene (L1014F), and the other mutation involves a leucine (TTA)-serine (TCA) transition in the same residue (L1014S) [7]. The L1014F mutation is only present at west of 10°W latitude in Africa, while L1014S is found in areas both west and east of 10°W, including Kenya [7].

Recently, a causal relationship between the high coverage of ITNs due to mass campaigns and the increase in the *kdr* frequency in *An. gambiae* s.s. has been reported [8], [9], [10]. The two point mutations are, therefore, important as indices of knockdown resistance in *An. gambiae* s.s., and periodical analyses of these mutations are essential for monitoring the development of pyrethroid resistance. *Anopheles arabiensis* Patton and *Anopheles funestus* Giles s.s., the former breeds in the same habitat as *An. gambiae* s.s. and the latter prefers to breed in permanent fresh waters with water weeds, are the second most common malaria vectors in Africa. The above point mutations, however, have been reported to be rare in *An. arabiensis* [8], [11], [12], [13], [14], [15] with the exception of 1 Ethiopian case [16], [17], and no such *kdr* mutation has been reported in *An. funestus* s.s. [18], [19], [20], [21]. On the contrary, evidence of metabolic resistance caused by the enhancement of P450 or glutathione S-transferase activity in these species has been reported [12], [20], [21].

The objective of the present study was to monitor the pyrethroid susceptibility in 3 major malaria vectors, *An. gambiae* s.s., *An. arabiensis*, and *An. funestus*, in a highly malaria endemic area in western Kenya and to elucidate the mechanisms of pyrethroid resistance in these species.

## Materials and Methods

### Study area

**Figure 1:**
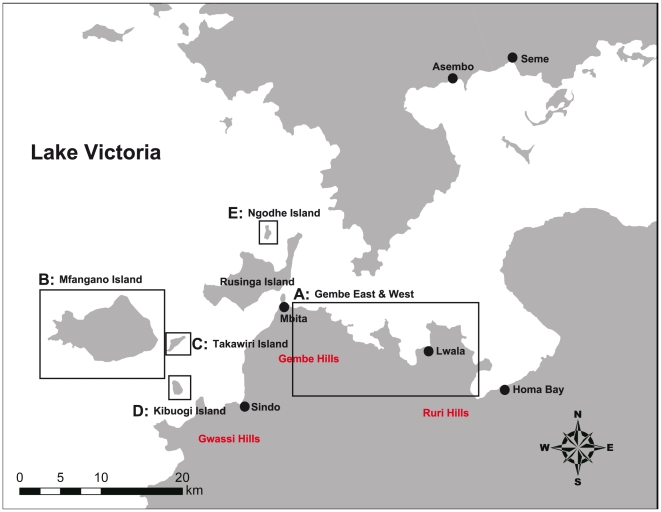
Map of study area (A, Gembe East & West; B, C, D, E, western islands).

Gembe East and West, Mbita Division, and 4 main western islands, Mfangano, Takawiri, Kibuogi, and Ngodhe, in the Suba district of the Nyanza province in western Kenya were used as study area (Figure 1). The Suba district has a population of 214,463 and an area of 1,063 km^2^ (2010). The district has 2 rainy seasons and is drier in the eastern part towards the Usao and Waondo locations and wetter towards the higher altitudes in the western parts of Gwassi hill and Mfangano Island. In the highlands, the rainfall ranges between 800–1900 mm per annum, while the lower area receives 800–1200 mm. The rainfall pattern in the area is bimodal, with the long rainy season occurring from March through May, and the short rainy season occurring in November and December. Malaria infection peaks briefly in June, following the long rains and more steadily between September and February [22]. The mean temperature is 25°C while the maximum temperature is 30°C, and humidity is relatively high.

The Suba district is one of the focal points identified as a high vector transmission area in Kenya, and more than 50% of the population is exposed to ≥40% *Pf*PR_2–10_ (*Plasmodium falciparum* parasite rate corrected to a standard age-range of 2 to less than 10 years old) [23]. A renewed effort to increase the coverage of effective preventative measures such as ITNs and combined vector control approaches have been adopted in this area. The Akado Medical Centre Project Mosquito Net and Power of Love Foundation in partnership with World Swim Against Malaria distributed 6,000 ITNs to children under five and pregnant women in the Gembe area. This increased the number of mosquito nets at least 3 fold, from 17% to 52% ITN coverage.

### Larval collection

**Figure 2:**
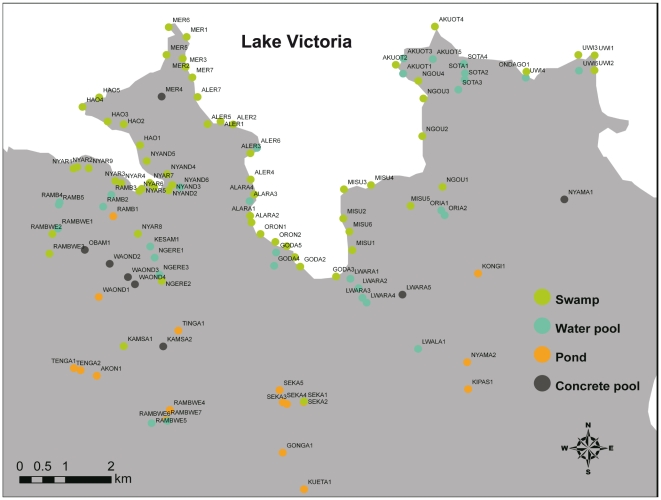
Mosquito breeding sites in Gembe East where larval collections were performed.

The typical breeding habitats of mosquitoes, such as small temporal pools, ponds, swamps, concrete pools, etc., in the Gembe East area were surveyed (Figure 2). Larvae were collected by dipping at each breeding site and brought to the laboratory for rearing and/or susceptibility testing. The geographical information, such as longitude, latitude, and elevation at each breeding site were recorded using a GPS unit. Larval collection was performed from May 5 to July 15, 2009.

### Adult collection and rearing of F1 progenies

**Figure 3:**
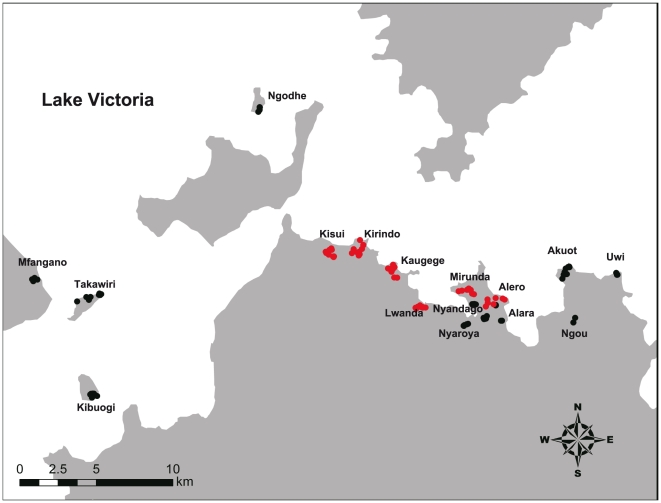
Adult collection sites. Black and red circles indicate aspirator and pyrethrum spray sheet collection sites, respectively.

Indoor mosquito collection was performed in the morning (7:00–10:00) by 2 conventional methods, namely, the pyrethrum spray sheet collection method (PSC) [24] (Gembe West and East area) and the aspiration with the battery-powered aspirator method (Cat. No. 2809C; BioQuip Products; Rancho Dominguez, CA, USA) (Gembe East and western islands area, Figure 3). After collection by aspiration, live blood-fed or gravid females were individually confined into a 20 ml glass vial with 2 ml of water and a strip of filter paper (ca 3×4 cm) was placed on the side of the vial to collect the eggs. Hatched larvae were reared with the lake water to obtain F1 larvae and adults for susceptibility testing. Larvae were fed with a mixture of powdered animal food (CE-2; Clea Tokyo, Japan, Inc.) and dried yeast (Ebios®; Mitsubishi Tanabe Pharma, Tokyo, Japan). Water cabbage (*Pistia stratiotes* L.) was added to the water for rearing *An. funestus* larvae. Adult collection was performed from October 1 to December 9, 2009 (Gembe East area), from February 1 to March 8, 2010 (Gembe East and western islands area), from April 19 to July 3, 2010 (Gembe East and West and western islands area), from September 13 to November 4, 2010 (Gembe East and West and western islands area), and from February 4 to March 5, 2011 (western islands area).

### Simplified knockdown bioassay using the mosquito larvae

The bioassay for the assessment of knockdown susceptibility was carried out on the day of collection by using the mosquito larvae obtained from the collection sites from which we could procure an adequate number of insects according to the simplified method previously described by Kawada et al. [25]. *D*-T_80_-allethrin was used for the test, since this chemical is one of the most conventional pyrethroid used for mosquito control and its high knock down activity is highly useful to the knock down bioassay. The larvae collected from each collection site were briefly identified on the day of collection, and fourth instar larvae of *An. gambiae* s.l., which occasionally might comprise a mixed population of *An. gambiae* s.s. and *An. arabiensis* individuals, were used for the susceptibility test. Each larva was individually placed in a glass vial with 20 ml of water. An emulsifiable concentrate of 90% *d*-T_80_-allethrin was diluted with water to obtain a 250-ppm solution. After releasing the larva, 32 or 8 µl of the solution was added in each vial to obtain a concentration of 0.4 and 0.1 ppm, respectively. At most, 20 larvae from each site were used for each concentration regime. Knockdown of the larvae was observed for 30 min. Larvae that sank to the bottom of the glass vial and could not swim, float, or were paralyzed were judged as knocked down larvae; the time to knockdown was recorded for each larva. After the test, each larva was placed in a 1.5-ml plastic vial containing ethanol solution for identification at a later time. The median knockdown times (KT_50_s), i.e., the time required for 50% knockdown, were scored according to the following 6 categories: 1, <5 min; 2, 5–10 min; 3, 10–15 min; 4, 15–20 min; 5, 20–30 min; and 6, >30 min. The susceptibility index was calculated as the product of the scores at 0.1 and 0.4 ppm. Thus, mosquito colonies with susceptibility index of 1 were considered to be the most susceptible, and those with susceptibility index of 36 were considered to be the least susceptible to *d*-allethrin. The larval bioassay was also performed for the larvae of F1 progeny to investigate the correlation in pyrethroid susceptibility between larvae and adults.

### Insecticide susceptibility test for adults

#### 1. Susceptibility test with WHO test tubes

Insecticide impregnated papers were prepared and adult susceptibility tests using WHO test tube kits were performed according to the WHO instructions (WHO/CDS/CPC/MAL/98.12). Insecticide impregnated papers, 0.75% permethrin (0.027 mg/cm^2^), 0.75% permethrin+0.75% piperonyl butoxide (PBO) (0.027 mg+0.027 mg/cm^2^), and 4% DDT (0.144 mg/cm^2^), were used for the tests. Female mosquitoes (1- to 3-day-old unfed F1 adults reared from the eggs of field collected females) were released in the WHO test tubes in order to expose them to the surface of an insecticide-impregnated paper for 1 hr; meanwhile, the time for knockdown was recorded. After exposure, the insects were transferred to the clean tube, fed with cotton soaked with 5% glucose solution, and mortality was recorded after 1 day. KT_50_ and average mortality for the mosquito colony were calculated. After the test, the insects were individually reserved in a 1.5-ml plastic vial with silica gel granules until detection of the *kdr* mutation.

#### 2. Susceptibility test by topical application

Technical grades of permethrin and PBO were used for the study. Female mosquitoes (1- to 3-day-old unfed F1 adults reared from the eggs of field collected females) were anesthetized briefly with carbon dioxide and were put on the metal cooling plate (Cool Plate NCP-2215; Nissin Scientific Corp, Tokyo, Japan), the surface temperature of which was maintained 4°C to maintain the anesthesia. A 0.25-µl acetone solution of the test chemical was topically applied to the dorsal mesothorax of 1- to 3-day-old female adults by using a Hand Microapplicator (Burkard Manufacturing Co., Ltd, Rickmansworth, UK). Treated insects were kept in a plastic cup, the bottom of which was lined with a filter paper, and fed with 5% glucose solution. Mortality was observed 24 h after treatment and LD_50_ (median lethal dosage) was calculated.

### Species identification

Mosquito larvae and collected adults were examined microscopically to distinguish *An. gambiae* s.l. and *An. funestus* s.l. from the other anophelines on the basis of the identification keys of Gillies and Coetzee [26]. Individual species within *An. gambiae* s.l. and *An. funestus* s.l. were identified using the multiplex polymerase chain reaction (PCR) method described by Scott et al. [27] and Koekemoer et al. [28].

### Detection of point mutations in the voltage-gated sodium channel

In order to verify the presence of point mutations at L1014 in *An. gambiae* s.s., *An. arabiensis*, and *An. funestus* s.s., PCR and direct DNA sequencing was conducted. The whole body of a larva or 2 to 3 legs of an adult were placed in a 1.5-ml PCR reaction tube. The sample was homogenized in a mixed solution of extraction solution (20 µl)+tissue preparation solution (5 µl) (REDExtract-N-AmpTM Tissue PCR Kit; SIGMA, St. Louis, MO, USA) for extraction of DNA. The solution was heated at 95°C for 3 min and neutralized. Initial fragment amplification was carried out using primers AGKF1(CATGATCTGCCAAGATGGAA) and AGKR1 (GTTGGTGCAGACAAGGATGA) for *An. gambiae* s.l.; and AFF1 (ACCAAGATCTGCCAAGATGG) and AFR1 (TGGTGCAGACAAGGATGAAG) for *An. funestus* s.s., respectively. The PCR mixture contained 4 µl of REDExtract-N-AmpTM ReadyMix (SIGMA), 0.5 µM of each primer, and 1 µl of the DNA template in a total volume of 10 µl. PCR was performed under the following conditions: 94°C for 3 min and 35 cycles of 94°C for 15 s, 55°C for 30 s, 72°C for 30 s, and 72°C for 10 min (for *An. gambiae* s.l.) or 94°C for 3 min and 35 cycles of 94°C for 15 s, 45°C for 30 s, 72°C for 30 s, and 72°C for 10 min (for *An. funestus* s.s.). The amplified fragments of the expected size were purified using ExoSAP-IT (USB Corporation, Cleveland, OH, USA) at a temperature of 37°C for 30 min and then 80°C for 15 min. DNA sequencing was carried out using primers Dg1 (TGGATHGARWSHATGTGGGAYTG) for *An. gambiae* s.l. and Dg3 (TGGATCGAATCCATGTGGGACTG) for *An. funestus* s.s., respectively. A BigDye Terminator v. 3.1 Cycle Sequencing Kit (Applied Biosystems Japan Ltd., Tokyo, Japan) was used for DNA sequencing according to the manufacturer's instructions. Direct DNA sequencing was performed by using the 3730 DNA Analyzer (Applied Biosystems). The electropherogram of the targeted amino acid replacement was analyzed by MEGA 4.0 public domain software (http://www.megasoftware.net/). All new data were deposited in GenBank (AB627099–AB627102).

### Statistical analysis

A digital map in shapefile format (Kenya-Boundaries, FAO Africover, http://www.africover.org/index.htm) was used for mapping of the collection sites. The geographical positions and the susceptibility index of larvae were plotted on the map by using ArcGIS 9.3 (ESRI Japan Corp, Tokyo, Japan.). Median knockdown times (KT_50_s) and lethal dosages (LD_50_s) were calculated by using the Bliss' probit method [29].

## Results

### Larval susceptibility against *d*-allethrin by simplified knockdown bioassay

Larvae of *An. gambiae* s.l. and *An. funestus* s.l. were collected from 82 breeding sites among the 117 sites surveyed. Larvae of *An. arabiensis*, which were collected in 74 sites, were common and dominant in the Gembe East area. The number of breeding sites where *An. gambiae* s.s. and *An. funestus* s.l. were collected, 4 and 4 sites respectively, were significantly fewer than those of *An. arabiensis*. Concrete pools, normally located in large permanent building site such as schools, seemed to be good breeding places for *An. arabiensis* larvae as well as the other culicine larvae (Table 1). A large number of *An. pharoensis* Theobald and a small number of *An. coustani* Laveran s.l. larvae were also collected at several sites as non-target species. Although quantitative sampling was not conducted and the sampling was mainly concentrated on the collection of late instar larvae for bioassay purposes, *An. arabiensis* larvae occupied 99% in the total number of collected larvae (1194), while the numbers of *An. gambiae* s.s. and *An. funestus* s.l. larvae were few (Table 2). The larval bioassay was, therefore, carried out solely using *An. arabiensis* larvae.

**Table 1:** Allelic frequency of L1014S mutations in Anopheline mosquito larvae collected in Gembe East, Mbita, Kenya.

Location	Breeding Place1)	Species - Allelic frequency of L1014S (AF %) and % of homozygous of L1014S (RR %)											
		*An. gambiae* s.s.			*An. arabiensis*			*An. funestus* s.s.			*An. rivulorum*		
		N	AF %	RR %	N	AF %	RR %	N	AF %	RR %	N	AF %	RR %
Nyaroya	SW, WP	-	-	-	124	0	0	-	-	-	-	-	-
Nyandago	SW, WP	-	-	-	78	0	0	-	-	-	1	0	0
Mirunda	SWL, CP	2	50	50	70	0	0	-	-	-	-	-	-
Hao	SWL	-	-	-	18	0	0	2	0	0	5	0	0
Alero	SW, SWL	-	-	-	75	0	0	-	-	-	-	-	-
Alara	SWL, WP	-	-	-	83	0	0	-	-	-	-	-	-
Orongo	SW, SWL	-	-	-	25	0	0	-	-	-	-	-	-
Godariyo	SW, SWL, WP	-	-	-	71	0	0	-	-	-	-	-	-
Misuri	SW, SWL	-	-	-	38	0	0	-	-	-	-	-	-
Ngau	SWL	-	-	-	26	0	0	-	-	-	-	-	-
Akuot	SWL, WP	-	-	-	59	0	0	-	-	-	-	-	-
Uwi	SWL, WP	-	-	-	69	0	0	-	-	-	-	-	-
Lambwe	SW, WP, PD	-	-	-	97	0	0	-	-	-	-	-	-
Kisamba	WP	1	100	100	17	0	0	-	-	-	-	-	-
Obambo	CP	1	100	100	14	0	0	-	-	-	-	-	-
Waondo	PD, CP	-	-	-	29	0	0	-	-	-	-	-	-
Kamsama	SW	-	-	-	20	0	0	-	-	-	-	-	-
Akonya	PD	-	-	-	18	0	0	-	-	-	-	-	-
Ngere	SW, WP	-	-	-	25	0	0	-	-	-	-	-	-
Tinga	WP, PD	-	-	-	20	0	0	-	-	-	-	-	-
Lwara	WP, CP	-	-	-	80	0	0	-	-	-	-	-	-
Oria	WP	-	-	-	16	0	0	-	-	-	-	-	-
Sota	WP	-	-	-	60	0	0	-	-	-	-	-	-
Rambwe	PD, WP	-	-	-	20	0	0	-	-	-	-	-	-
Nyamaji	CP	-	-	-	20	0	0	-	-	-	-	-	-

1) SW, swamp; SWL, swamp near lake shore; WP, small water pool; PD, pond; CP, concrete pool.

**Table 2:** Collection Data of Anopheline mosquitoes in Gembe East, Gembe West, and western islands, Kenya.

Sampling Method	Collection Place	Date of Collection	Test Performed (generation used)	Collected species (figures in parenthesis are No. of females oviposited)					
				*An. arabiensis*		*An. gambiae* s.s.		*An. funestus* s.s.	
				F	M	F	M	F	M
Larval Collection (Dipping)	Gembe East	May 5–July 15, 2009	Simplified Knockdown Assay (F0) Detection of *kdr* (F0)	1182		4		2	
				( - )		( - )		( - )	
Adult Collection (Aspiration)	Gembe East	Oct 1–Dec 9, 2009	WHO Tube Test for Permethrin and DDT (F1) Detection of *kdr* (F0 & F1)	67	34	1	0	14	0
				(40)		(0)		(10)	
	Western islands	Sept 13–Nov 4, 2010 Feb 4–Mar 5, 2011		0	0	4	0	0	0
				( - )		(3)		( - )	
Adult Collection (Aspiration)	Gembe East	Feb 1–Mar 8, 2010	WHO Tube Test for Permethrin and PBO (F1) Detection of *kdr* (F0 & F1)	30	7	1	0	74	11
				(17)		(0)		(32)	
	Western islands			4	1	28	15	1	0
				(1)		(5)		(0)	
Adult Collection (Aspiration) Larval Collection (Dipping)	Gembe East	Apr 19–July 3, 2010	Topical Application for Permethrin and PBO (F1) Detection of *kdr* (F0 & F1)	671)	141)	8	3	686	241
				(35)		(1)		(137)	
	Western islands			6	0	81	12	3	0
				(4)		(33)		(0)	
Adult Collection (Pyrethrum Spray Sheet)	Gembe East	Apr 19–July 3, 2010 Sept 13–Nov 4, 2010	Detection of *kdr* (F0)	0	-	0	-	1922)	-
				( - )		( - )		( - )	
	Gembe West			165	-	4	-	2632)	-
				( - )		( - )		( - )	

1) Additionally, larvae were collected in Nyaroya and emerged 189 female adults were used for topical application test.,

2) Additionary, 59 females and 16 males, and 7 females of *An. rivurolum* were collected in Gembe East and Gembe West, respectively.

**Figure 4:**
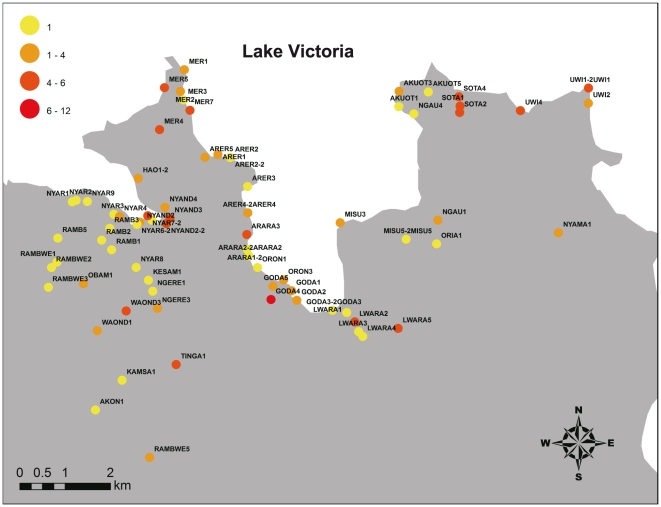
Distribution of larval susceptibility index for *An. arabiensis* collected in Gembe East area.

Figure 4 shows the distribution of the susceptibility index in field-collected *An. arabiensis* larvae against *d*-allethrin in Gembe East area. Almost all the larval colonies of *An. arabiensis* showed low susceptibility indices (<6) except for 1 colony collected in Godariyo (Site ID GODA4) of which the index was 12. Furthermore, not a single point mutation at L1014 was observed in all larval colonies of *An. arabiensis* (Table 1). On the other hand, however, among the 4 *An. gambiae* s.s. larvae collected at Mirunda, Kisamba, and Obambo, 3 larvae had homozygous L1014S mutations.

### Adult susceptibility against permethrin and DDT by WHO tube test

F1 progenies of mosquitoes collected from October 1 to December 8, 2009 in the Gembe East area and those collected from September 13 to November 4, 2010 and February 4 to March 5, 2011 in the western islands were used for the test (Table 2). F1 progenies from 22 female *An. arabiensis* collected in 9 different houses, 4 female *An. gambiae* s.s. collected in different houses, and 7 female *An. funestus* s.s. collected in 5 different houses were tested. The data for F1 progenies of females collected from the same house were mixed for the calculation of KT_50_ and mortality. Additionally, one laboratory colony of *An. gambiae* s.s. collected in Lwanda, Mbita in 2002 that has been maintained in the Thomas Odhiambo campus of the International Center of Insect Physiology and Ecology (ICIPE) was also used as a reference. The allelic frequency and the rate of homozygous L1014S point mutations in this colony were relatively low (6.3% and 0%). Table 3 shows the insecticide susceptibility of F1 larvae and female adults by the simplified larval knockdown assay and WHO tube test. Not a single point mutation at L1014 was observed in all F1 colonies of *An. arabiensis* and *An. funestus* s.s. Adult susceptibility of both species against DDT seemed to be high, showing a >80% mortality and a KT_50_ of <60 min. On the other hand, the susceptibilities against permethrin were relatively low in both species. In total, 7 (77.8%) and 3 (60%) colonies showed KT_50_s of >60 min and all colonies showed mortalities of <80% in *An. arabiensis* and *An. funestus*, respectively. Larval susceptibility indices were low (<12) in almost all colonies except for 1 colony in *An. funestus* s.s. Although the sample sizes were small, there seemed to be no correlation between the larval susceptibility indices and adult mortality against permethrin in *An. arabiensis*. On the other hand, *An. gambiae* s.s. colonies, except for laboratory colony (ICIPE) and one colony with no homozygous L1014S collected in Nyandago (NYAND 8), showed low mortality and a KT_50_s of >60 min against both permethrin and DDT, indicating that the positive correlation with larval susceptibility for these colonies (susceptibility indices were >30).

**Table 3:** Susceptibility against permethrin and DDT of F1 larvae and female adults produced by individual blood-fed females collected in houses in Gembe East, Mbita and western islands, Kenya.

Species	House ID2) ^,^ 3)	F1 larvae		F1 adults (WHO Tube Test)						
		N	Susceptibility Index (*d*-allethrin)	Permethrin 0.75%			DDT 4%			RR %1)
				N	KT_50_ (min.)	% Mortality	N	KT_50_ (min.)	% Mortality	
*An. arabiensis*	NYAND 6	24	6	20	45–60	75.0	10	20–30	100	0
	NYAND 8	26	6	26	>60	48.0	17	29.1	100	0
	NYAND 11	30	6	30	>60	30.0	25	34.2	100	0
	NYAND 13	10	6	4	>60	75.0	-	-	-	0
	NYAR 1	-	-	13	>60	23.1	12	30–45	100	0
	NYAR 3	10	6	13	47.5	76.9	10	28.1	100	0
	NYAR 5	74	12	45	>60	64.4	33	41.0	100	0
	NYAR 6	14	6	10	>60	50.0	10	30–45	100	0
	NYAR 8	10	12	10	>60	44.4	10	27.3	100	0
*An. gambiae* s.s.	ICIPE4)	40	2	26	38.1	100	21	30.2	100	0
	NYAND 8	-	-	3	>60	33.3	3	45–60	100	0
	MFA 1	20	36	32	>60	50.0	25	>60	56.0	100
	MFA 6	14	36	27	>60	0	28	>60	13.8	100
	TAKA 4	8	30	15	>60	14.3	15	>60	60.0	100
*An. funestus* s.s	NYAND 11	-	-	2	30–45	50.0	-	-	-	0
	NYAR 1	10	6	16	>60	26.7	12	32.6	83.3	0
	NYAR 3	-	-	12	>60	54.5	1-	30–45	100	0
	NYAR 7	20	36	8	30.6	62.5	4	20.8	100	0
	NYAR 8	-	-	1	>60	0	-	-	-	0

1) % of homozygous L1014S mutation,

2) Collection site - House No.,

3) NYAND, Nyandago; NYAR, Nyaroya; MFA, Mfangano; TAKA, Takawiri,

4) Laboratory colony reared in ICIPE since the collection at Lwanda, Mbita in 2002.

### Synergism of permethrin and PBO by WHO tube test

F1 progenies of mosquitoes collected from February 1 to March 8, 2010 in the Gembe East area and western islands were used for the test (Table 2). F1 progenies from 18 female *An. arabiensis* collected in 12 different houses, 5 female *An. gambiae* s.s. collected in 4 different houses, and 4 female *An. funestus* s.s. collected in 4 different houses were tested. The data for F1 progenies of females collected from the same house were mixed for the calculation of KT_50_ and mortality. The results are shown in Table 4. All colonies of the 3 species showed a KT_50_s of >60 min and 17 (94.4%) colonies of *An. arabiensis* and all colonies of *An. gambiae* s.s. and *An. funestus* s.s. showed mortalities of <80% when they were exposed to permethrin 0.75% impregnated paper, indicating the high resistance of these species to permethrin. On the contrary, faster knockdown and higher mortality were observed in the test when female adults of the same colonies were exposed to permethrin+PBO 0.75/0.75% paper. All colonies and 3 (75%) colonies of *An. arabiensis* and *An. funestus* s.s. showed KT_50_s of <60 min, and 14 (77.8%) colonies of *An. arabiensis* and 2 (50%) colonies of *An. funestus* s.s. showed mortalities of >80% in this case, indicating that permethrin+PBO showed high synergism to the above 2 species. The reaction of *An. gambiae* s.s., on the other hand, was different from those of the other species. Although some improvement in mortality was observed (>80% mortality in 2 of 5 colonies), there was no improvement in knockdown activity by the synergist in this species. Furthermore, *An. gambiae* s.s. colonies had L1014S point mutations at high frequency, while not a single point mutation at L1014 was observed in all F1 colonies of *An. arabiensis* and *An. funestus* s.s.

**Table 4:** Susceptibility against permethrin and permethrin/PBO of F1 female adults produced by individual blood-fed females collected in houses in Gembe East, Mbita, and western islands, Kenya.

Species	House ID2) ^,^ 3)	WHO Tube Test (F1 adult)						
		Permethrin 0.75%			Permethrin/PBO 0.75/0.75%			RR %4)
		N	KT_50_ (min.)	% Mortality	N	KT_50_ (min.)	% Mortality	
*An. arabiensis*	NYAR 3	26	>60	47.6	23	24.8	90.9	0
	NYAR 5	14	>60	25.0	15	26.0	100	0
	NYAR 10	25	>60	24.0	32	26.8	93.8	0
	NYAND 8	15	>60	0	16	18.0	100	0
	AKU 1	12	>60	30.0	13	16.3	100	0
	AKU 2	14	>60	7.1	17	42.1	70.6	0
	AKU 3	12	>60	8.3	13	20.3	84.6	0
	AKU 4	12	>60	0	12	20.2	100	0
	NGOU 2	3	>60	0	3	30–45	66.7	0
	UWI 2	12	>60	16.7	13	31.3	76.9	0
	TAKA 2	2	>60	0	3	24.5	33.3	0
	KIBU 1	17	>60	55.6	18	18.2	100	0
*An. gambiae* s.s.	ICIPE1)	26	38.1	100	20	17.0	100	0
	TAKA 1	25	>60	8.0	28	>60	84.6	100
	KIBU 2	3	>60	0	5	>60	0	75
	MFA 3	15	>60	46.7	12	>60	41.6	91.7
*An. funestus* s.s.	NYAND 6	2	>60	0	2	15–20	100	0
	NYAR 3	11	>60	30.0	11	28.3	54.5	0
	NYAR 4	1	>60	0	2	>60	50.0	0
	NYAR 10	4	>60	0	5	36.7	100	0

1) Laboratory colony reared in ICIPE since the collection at Lwanda, Mbita in 2002,

2) Collection site - House No.,

3) NYAR, Nyaroya; NYAND, Nyandago; AKU, Akuot, TAKA, Takawiri; KIBU, Kibuogi; MFA, Mfangano,

4) % of homozygous L1014S mutants.

### Synergism of permethrin and PBO by topical application

F1 progenies of mosquitoes collected and female adults emerged from the larvae collected in Nyaroya from April 19 to July 3, 2010 in the Gembe East area and western islands were used for the test (Table 2). By aspirator collections of adult mosquitoes in the Gembe East area, *An. arabiensis*, *An. gambiae* s.s., and *An. funestus* s.s. were collected in 22 houses. Finally, *An. gambiae* s.s. were collected in 22 houses in the 4 western islands, while the other 2 species were few. F1 progenies from the same village were mixed and used for the topical application test. For evaluation of a synergism, a mixed solution of PBO and permethrin, in which the concentration of PBO was fixed (1.25 µg/female), was topically applied to the females of the same colony.

Table 5 shows the LD_50_s of permethrin for village-based groups of F1 progenies of *An. arabiensis*, *An. gambiae* s.s., and *An. funestus* s.s. Reduction in LD_50_s by PBO treatment was prominent in *An. arabiensis* and *An. funestus* s.s., and the synergic effects (Ratio P/B) for both species were >20. The synergic effect of PBO in *An. gambiae* s.s., on the other hand, was lower than those in the above 2 species (<10). *Anopheles gambiae* s.s. colonies had L1014S point mutations at high frequency (70.4–100%), while not a single point mutation at L1014 was observed in all F1 colonies of *An. arabiensis* and *An. funestus* s.s.

**Table 5:** Susceptibility against permethrin and permethrin/PBO of F1 female adults produced by individual blood-fed females collected in houses in Gembe East, Mbita, and western islands, Kenya by topical application.

Species	Location1)	Permethrin (P)				Permethrin+PBO (1.25 µg) (B)				% RR4)	RatioP/B
		LD_50_ (µg/female)	95% C.L.	Slope	N	LD_50_(µg/female)	95% C.L.	Slope	N		
*An. arabiensis*	Nyandago	0.017	(0.0097–0.026)	3.0	51	0.00039	-	1.2	30	0	44
	Nyaroya2)	0.092	(0.063–0.16)	2.0	99	0.0042	(0.0023–0.012)	2.3	90	0	22
	Nyaroya	0.023	(0.060–0.045)	1.3	67	0.00059	(0.00027–0.00094)	1.8	75	0	39
	Alero/Alara	0.024	(0.0063–0.046)	2.1	36	0.00042	-	1.1	27	0	57
	Akuot	0.011	(0.00041–0.017)	2.3	30	0.00032	(0.000066–0.00075)	1.1	28	0	34
	Mfangano	0.020	-	2.8	35	0.00029	-	1.7	38	0	69
*An. gambiae* s.s.	ICIPE3)	0.0030	(0.0023–0.0035)	2.5	182	0.00062	(0.00024–0.00097)	1.1	151	0	4.8
	Ngodhe	0.036	(0.022–0.057)	1.9	66	0.0047	(0.0026–0.0088)	1.4	80	87.6	7.7
	Takawiri	0.057	(0.015–0.91)	0.89	36	0.015	(0.0074–0.023)	4.3	41	92.5	3.8
	Mfangano	0.15	-	1.0	54	0.020	-	3.8	62	70.4	7.5
	Kibuogi	0.078–0.16	-	-	36	0.044	(0.012–0.094)	2.4	40	100	1.8–3.6
*An. funestus* s.s.	Nyandago	0.018	-	3.4	31	0.00031	-	2.3	17	0	58
	Nyaroya	0.012	(0.0081–0.021)	3.9	33	0.00051	-	2.3	26	0	24

1) Location where parental females were collected,

2) Collected as larvae,

3) Laboratory colony reared in ICIPE since the collection at Lwanda, Mbita in 2002,

4) % of homozygous L1014S.

### Allelic frequency of L1014S mutations in Anopheline mosquitoes collected in the study area

The allelic frequencies and percentages of homozygous L1014S mutations in *An. arabiensis*, *An. gambiae* s.s., *An. funestus* s.s., and *An. rivulorum* collected in Gembe East and West and the western islands are shown in Table 6. The West African type mutation, L1014F, was not detected in our study. Not a single L1014S mutation was detected in *An. arabiensis* (208 females and 77 males) and *An. funestus* s.s. (613 females and 108 males). Significantly high allelic frequencies (>90%) and percentages of homozygous L1014S mutations (>80%), on the contrary, were detected in *An. gambiae* s.s. High allelic frequency in the L1014S mutation seemed to be equally detected in *An. gambiae* s.s. collected in the inland areas as well as those collected in the islands, even though the number of collection was small.

**Table 6:** Allelic frequency of L1014S mutations in Anopheline mosquito adults collected in Gembe East and Gembe West, Mbita, and western islands, Kenya.

Species	Location	Female			Male		
		N	AF %1)	RR %2)	N	AF %	RR %
*An. arabiensis*	Nyandago	53	0	0	27	0	0
	Nyaroya3)	50	0	0	23	0	0
	Nyaroya	76	0	0	22	0	0
	Alero/Alala	5	0	0	2	0	0
	Akuot	12	0	0	1	0	0
	Ngou	2	0	0	-	-	-
	Uwi	1	0	0	1	0	0
	Kirindo	10	0	0	-	-	-
	Kaugege	50	0	0	-	-	-
	Lwanda	104	0	0	-	-	-
	Mfangano	4	0	0	1	0	0
	Takawiri	5	0	0	-	-	-
*An. gambiae* s.s.	ICIPE4)	405)	6.3	0	-	-	-
	Nyandago	2	100	100	1	0	0
	Nyaroya	2	0	0	-	-	-
	Akuot	6	100	100	2	100	100
	Kaugege	1	100	100	-	-	-
	Lwanda	2	50	50	-	-	-
	Takawiri	40	93.8	92.5	6	66.7	66.7
	Ngodhe	35	92.9	91.4	3	100	100
	Mfangano	24	95.9	91.7	18	91.7	83.3
	Kibuogi	8	93.8	87.5	-	-	-
*An. funestus* s.s.	Nyandago	221	0	0	93	0	0
	Nyaroya	164	0	0	12	0	0
	Mirunda	95	0	0	-	-	-
	Akuot	12	0	0	1	0	0
	Ngou	5	0	0	-	-	-
	Alero/Alala	117	0	0	3	0	0
	Kaugege	105	0	0	-	-	-
	Lwanda	144	0	0	-	-	-
	Kirindo	10	0	0	-	-	-
	Kisui	1	0	0	-	-	-
	Mfangano	1	0	0	-	-	-
	Takawiri	2	0	0	-	-	-
*An. rivulorum*	Nyandago	54	0	0	15	0	0
	Nyaroya	4	0	0	1	0	0
	Mirunda	1	0	0	-	-	-
	Lwanda	6	0	0	-	-	-
	Kirindo	1	0	0	-	-	-

1) Allelic frequency of L1014S mutations,

2) % of homozygous L1014S mutations,

3) Collected as larvae,

4) Laboratory colony reared in ICIPE since the collection at Lwanda, Mbita in 2002,

5) Tested with larvae.

## Discussion

By the collection data in the present study, *An. gambiae* s.s. dominated in the 4 western islands, while *An. arabiensis* dominated in the inland areas of Gembe East and West. In the study area, however, *An. gambiae* s.s. had been a dominant species since the end of 20th century [30]. Recently, Bayoh et al. [10] reported the same inequality in the 2 species in western Kenya, finding that the frequency of *An. gambiae* s.s. varied by site with frequencies of <15% at sites west of Kisumu and along the lakeshore (Asembo and Kisian) but frequencies of >80% at sites further from the lakeshore (Busia, Bungoma, Kakamega, and Malaba). The authors also reported that the frequency of *An. gambiae* s.s. declined at 2 sites along the lakeshore (Asembo and Kisian) after 1996 in association with the rollout of LLINs. The study area of the present report was located in the south of the above area on the opposite side of Lake Victoria. Similar unknown ecological, meteorological, or artificial factors, therefore, seem to relate to these phenomena. Mathias et al. [9] also stated that the East African *kdr* allele (L1014S) coincidentally increased in frequency during the past decade in *An. gambiae* s.s. in western Kenya, most of which are homozygous *kdr* allele, as household ownership of insecticide-treated bed nets increased regionally. The same correlation between the increase in L1014S frequency and increased use of ITN was reported by Stump et al. [8]. In our study, the allelic frequency of L1014S mutation in the *An. gambiae* s.s. colony collected in 2002 was found to be low (6.3%), while the recent wild colonies collected in several locations had high frequency (>90%). Our findings not only support the above previous reports concerning the increase in *kdr* frequency during the past decade, but they also clarify the lower contribution of the P450-related metabolic resistance factors in *An. gambiae* s.s. since the synergistic effect of PBO was significantly lower in *An. gambiae* s.s. than the other 2 species (Tables 4 and 5).

 In contrast, P450-related pyrethroid resistance was found to be widespread in both *An. arabiensis* and *An. funestus* s.s. in the study area. According to the few reports on the evaluation of insecticide efficacy against *An. gambiae* s.l. by the topical application method, the LD_50_ of permethrin in susceptible *An. gambiae* s.s. is thought to be 0.0001 to 0.001 µg/female [31], [32], [33]; that finding corresponds to the LD_50_s of permethrin in *An. arabiensis* and *An. funestus* s.s. in the present report when it was synergized by PBO, indicating that resistance factors in the above 2 species are mainly due to P450-related enhanced metabolism (Table 5). Lack of L1014S mutation in these species also supports the above facts (Table 6). On the contrary, larval susceptibilities to *d*-allethrin in *An. arabiensis* and *An. funestus* s.s. were relatively high and did not correlate to adult susceptibility to permethrin (Table 3), suggesting that different metabolic mechanisms play a role in the larval and adult stages. The correlation between adult and larval susceptibility against pyrethroids seems to be common [31], [34], especially when insects have homozygous *kdr* mutations at high frequency [25], [35]. This is, however, not always true for all cases, since mosquitoes may develop different resistance mechanisms through different metabolic pathways in the larval and adult stages. A similar case was previously reported regarding the malathion resistance of *An. arabiensis* in Sudan [36], in which the authors attributed the absence of larval resistance to the house spraying as the major source of selection pressure rather than agricultural spraying. The L1014S or L1014F point mutations have been reported to be rare in *An. arabiensis* [8], [11], [12], [13], [14], [15] with the exception of one Ethiopian case [16], [17]. Not a single L1014S mutation in *An. arabiensis* was found in Chad [11], Zimbabwe [13], or Malawi [14]; only 1 heterozygous L1014S mutation was found of 572 samples in Asembo, Kenya [8] and of 54 samples in Ahero, Kenya [12]; and 9 (0.04%) homozygous and 4 (0.02%) heterozygous L1014S mutations were reported in 243 *An. arabiensis* in Uganda [15]. The pyrethroid resistance in *An. arabiensis* in Ethiopian cases [16], [17] is unique and exceptional since the West African type L1014F mutations were found at high frequency, whereas East African type mutations (L1014S) were absent. The authors suggested that the high frequency of such *kdr* mutations might be attributed to the long intensive use of DDT in indoor residual spraying (IRS) for malaria control and/or to the illegal extensive use of DDT for the control of agricultural pests. In contrast, the present study shows a lack of cross-resistance between DDT and permethrin in *An. arabiensis* (Table 3). In Nyanza province, dieldrin was reported to be administered mainly through aerial spraying especially for tsetse fly control [37], while the organized intensive spray of DDT for mosquito control was not performed in the 1970s and 1980s (Mwatele et al. personal communication), and no IRS has been administered since then. Therefore, it might not be possible to attribute the DDT participation to the causality of metabolic pyrethroid resistance in *An. arabiensis* in the study area, suggesting that the different selection pressure resulted in a different resistance mechanism from those in the same species in Ethiopian cases. The cross-resistance between permethrin and DDT in *An. gambiae* s.s. in the study area is thought to be attributed to the high frequency of *kdr* mutation, which might be selected by the frequent exposure to ITNs.

Recently, high levels of pyrethroid resistance in *An. funestus* s.s. have been reported in Uganda [18], Ghana [19], and Mozambique [20], [21]. No *kdr* mutation has been reported in the above reports, while evidence of metabolic resistance caused by the enhancement of P450 or glutathione S-transferase activity in these species has been reported. Morgan et al. [18] stated that their findings on pyrethroid/DDT resistance in *An. funestus* s.s. in Uganda were the first reports from East Africa, indicating that resistance in *An. funestus* s.s. might be more widespread in Africa than previously assumed. No pyrethroid resistance in *An. funestus* s.s. has been reported in Kenya [38], and our study may be the first report in Kenya.

*Anopheles gambiae* s.s., *An. arabiensis*, and *An. funestus* s.s. are the most widely distributed vectors in Kenya [39] and are thought to be the most important species for malaria transmission. It is noteworthy that the above species in the same area are found to be resistant to pyrethroids by their unique resistance mechanisms. Furthermore, it is interesting that 2 different resistance mechanisms have developed in the 2 sibling species in the same area individually, indicating the absence of gene flow between these species. The difference in biological characteristics between the 2 species might be hypothesized to explain the above. The extent of selection pressure might depend on the environmental difference in the major breeding sites treated with agricultural pesticides and on the difference in anthropophily in insecticides for indoor spray or ITN. Anthropophilic *An. gambiae* s.s. might have a greater chance to be exposed to insecticides for indoor use than *An. arabiensis* and *An. funestus* s.s., which are more zoophilic than *An. gambiae* s.s. [40]. Similarly, Bayoh et al. [10] concluded that the historical decline in *An. gambiae* s.s. populations associated with intensive distribution of ITNs at sites west of Kisumu and along the lakeshore might partly be attributable to the above difference in the anthropophily of mosquitoes. *An. funestus* s.s. is more anthropophilic than *An. arabiensis* [40] and this species might also have experienced the selection by LLITNs than *An. arabiensis*. Another main difference among three species comes probably from their breeding sites which are more polluted by agricultural pesticides for *An. gambiae* s.s. and *An. arabiensis* but less for *An. funestus*. The present study also suggests that the combinational use of a synergist such as PBO might reduce the resistance levels of *An. arabiensis* and *An. funestus* s.s. to their original susceptibility levels. Simplistic and intensive use of such combinations, however, might trigger another resistance mechanism.

 Insecticides still provide the most promising countermeasures for controlling malaria as well as other arthropod-borne diseases. At the global level, 547 tons of DDT, 39 tons of organophosphates, 23 tons of carbamates, and 41 tons of pyrethroids are used annually for indoor residual spraying against malaria vectors [6]. The average total amount of pyrethroids used annually between 2003 and 2005 was 161 tons, which is 36% of the total insecticide consumption if the amount of DDT, which is exclusively used in African countries, is excluded. Among pyrethroids that are used for vector control, 98.7% comprise photostable pyrethroids such as μ-cypermethrin, μ-cyhalothrin, cyfluthrin, cypermethrin, deltamethrin (type II), etofenprox, bifenthrin, and permethrin (type I) [6]. Vector resistance against these pyrethroids has been a worldwide concern. It is expected that the use of photo-unstable knockdown agents such as spatial repellents, which effectively interfere with disease transmission without causing any selection pressure to insect populations, will be reconsidered [41]. Additionally, new self-protection measures using exito-repellent type I pyrethroids are of great interest as substitutional or supplemental techniques for bio-rational vector control measures in the future. The most popular and long-standing formulations using pyrethroids are mosquito coils, mosquito mats, and liquid vaporizers. Pyrethroids belonging to the knockdown agent group, such as allethrin, pyrethrin, and prallethrin, are used in these formulations. In particular, *d*-allethrin still continues to be used in these types of formulations. Further, mosquitoes have developed minimum physiological resistance to these pyrethroids due to the low selection pressure. Pyrethroids belonging to the knockdown agent group have been successfully used worldwide for a long period as a spatial repellent. Spatial repellency will not induce any pyrethroid resistance since it has low lethal activity on the affected insects and causes less selection pressure on insect populations. The discovery of the phenoxybenzyl alcohol moiety accelerated the development of photostable pyrethroids that could be used for outdoor use, including agricultural purposes. These “second generation” pyrethroids have been used worldwide as good vector control agents with various application techniques, such as residual spraying, ULV spraying, and LLIN. However, photostable and highly effective pyrethroids might accelerate the development of pyrethroid resistance in mosquito populations. Photostable pyrethroids consist of 2 structurally different types of chemicals according to the presence of μ-cyano moiety, type I (permethrin, etofenprox, etc.) and type II (deltamethrin, lambda-cyhalothrin, cypermethrin, etc.). Siegert et al. [42] reported that the Olyset® Net, slow-released polyethylene formulation containing 1000 mg of permethrin per m^2^, fitted over the human hand reduced landing attempts and elevated flight frequency, resulting in little mortality, while mosquito landing attempts on the PermaNet®, containing 55 mg of deltamethrin per m^2^, under the same conditions were sustained longer and caused greater mortality than the Olyset® Net. The authors concluded that the optimal LLIN formulation should maximize taxis or kinesis (“engagement”) so as to maximize mortality. This appears to be important for an effective control of the mosquito population. The high lethal pyrethroids, however, might accelerate the development of resistance. The exito-repellency of slow-released permethrin, on the contrary, might reduce the human-vector contact and blood feeding success [43]. In fact, there was no difference between Olyset® Net and PermaNet® in the field efficacy as measured by blood feeding rate [44]. The positive use of exito-repellency of slow-released pyrethroids, therefore, might lead bio-rational vector control with the maximum reduction of mosquito biting and minimum risk of resistance.
